# Recovery of Body Awareness After Stroke: An Observational Study

**DOI:** 10.3389/fneur.2021.745964

**Published:** 2021-11-29

**Authors:** Ines Serrada, Brenton Hordacre, Susan Hillier

**Affiliations:** Allied Health and Human Performance, Innovation, Implementation and Clinical Translation in Health, University of South Australia, Adelaide, SA, Australia

**Keywords:** physiotherapy, stroke, rehabilitation, sensation, body awareness

## Abstract

**Background:** Body awareness (BA) is a process that involves sensory awareness originating from the body's physiological states, processes and actions, and is shaped by one's attitudes, perceptions, beliefs and experience of social and cultural context. Impairments in body awareness after stroke are believed to be common and may be an important influence on recovery outcomes. However, recovery of body awareness is poorly understood and receives little consideration in rehabilitation.

**Aims:** To investigate if body awareness changes over time following stroke; and identify if body awareness after stroke is associated with sensation, motor impairment, self-efficacy and quality of life.

**Methods:** An exploratory longitudinal observational study was performed. Participants with a stroke diagnosis and associated motor impairment were recruited from an acute stroke unit. An assessment battery consisting of sensory and motor impairment and function, body awareness, self-efficacy and quality of life measures were used at baseline, 1, 3 and 6 months.

**Results:** A total of 105 people with stroke were recruited. Most recovery in sensation and body awareness occurred within the first month after stroke (all *p* < 0.01). Sensation and body awareness were correlated with other clinical outcomes (motor impairment, self-efficacy and quality of life), demographics, and stroke specific clinical characteristics (all *p* < 0.01).

**Conclusions:** This is the first study to track recovery of body awareness after stroke and investigate the relationship it may have in recovery of sensation, motor impairment and function, self-efficacy and quality of life. Further research is now warranted to continue investigation of body awareness and to develop effective stroke-specific assessment and intervention strategies.

## Introduction

Body awareness is considered an interactive process that includes awareness of the body's *physiological states, processes (including pain and emotion) and actions (including movement), and is shaped by an individual's attitudes, perceptions, beliefs and social/cultural context experiences* (1) (p.2). The nature of impairments post-stroke would suggest that body awareness may likely be impacted in stroke survivors how this has received little attention in the literature.

Awareness has been proposed to develop from a **body schema** (unconscious representation of the position of the body in space plus the position of sensation on the body surface) (2–31), and **body image** (a conscious representation of one's self) (2, 3, 7–10, 13, 17, 19, 32–34).

Considering recent literature, the triadic model has been proposed to further explain the nature and properties of body representations. The triadic model retains the dyadic (schema and image) notion however subdivides body image into two further representations: body structural descriptions and body semantics. Body structure describes a topological map primarily derived from visual input but also somatic perception. It provides a structural description of the relationships between body parts boundaries, proximity and relative position (2, 9, 12, 15, 25–28, 35–38). Body semantics describes the relationship between words and meaning and represents semantic and lexical information about the body (including functions of body parts, associations between body parts and objects, and body part names) (9, 12, 15, 25–28, 35–40).

The importance of body awareness lies in its' role in constantly monitoring, updating and providing feedback about the position and movement of one's body through space. It is also the main process used in integrating information for perception, decision making and action, making accurate body information essential for the precise control of movements (10, 41). The neuroanatomical basis influencing body awareness is understood to include an integrated system of brain regions and functional networks. The main regions within the somatosensory network (and important for body schema) are found primarily in the parietal cortex (SI and SII) as well as the thalamus, insula and cerebellum (50, 75, 94). A more distributed network, including attention and visual networks, is involved in the conscious processing of somatosensory information (50, 75, 95). Information processing of sensation for perception, and sensation for action, is described to involve both parallel and serial processing (75). It is important to note that all senses (exteroception and interoception) feed into the representation/s. Furthermore, body image involves affective and memory input from the limbic system and the semantic and lexical aspects require input from the language and spatial areas of the parietal lobes in their respective hemispheres.

Intact body awareness is thought to be a major factor that supports motor function and recovery of individuals following stroke (10, 41). One in two people experience impairments in sensation and perception after a stroke which interrupts the representation of the body that is held in the brain (42) and has a profound impact on an individual's body awareness (43–46). Altered motor and sensory cortical processing leads to inaccurate body information which can manifest in many different ways such as altered perception of limb size, position, shape or weight. This impairs the precision and control of one's movements (including postural control, dynamic balance, coordination) and the individual's ability to explore the immediate environment safely (41, 43–45). Subsequently it affects one's functional abilities, execution of daily activities and quality of life (35, 41, 47–49), making simple actions such as preparing breakfast, taking a shower or going for a walk challenging (10, 40). Further, reduced body awareness often interferes with the duration of rehabilitation and discharge destination (43, 45, 50).

Emphasising further the important role of body awareness in stroke recovery, body awareness training has been linked to positive rehabilitation outcomes, particularly with balance and mobility (51–53). However, we currently have little understanding of body awareness during stroke recovery or whether it is important for enabling behavioural restitution. While much work has been focussed on initial motor impairment, structural damage and the neurobiological course of the recovery process (54), little attention has been directed to body awareness (43). Indeed, from sensory and motor impairment studies, research suggests recovery is most marked within the first 3 months after stroke, although ongoing recovery can be observed at 6 months and later (54–56). In particular, evidence from sensory rehabilitation studies have indicated the potential for marked recovery from months to years after stroke (43), and body awareness similarly may continue to evolve over the first 2 years (7, 49). There is some suggestion that individuals within the first 2–6 months direct their attention toward the way their body functions and try to find new ways to manage daily activities and actions. Subsequently from 6 to 12 months the focus shifts to forming an understanding and acceptance of their bodily changes (7).

The purpose of this study was to first investigate if body awareness is impaired after a stroke and if it recovers over time, and second, identify if body awareness is associated with sensation, motor impairment, self-efficacy and quality of life. It was hypothesised that body awareness will initially be impaired after stroke, improve within the first few months and will be associated with improvements in motor, sensory and quality of life measures.

## Methods

### Study Design

An exploratory, prospective, longitudinal, observational study was conducted and reported using the STROBE guidelines. Recruitment for this trial commenced September 2017 and concluded in June 2019. Ethical approval was obtained from the University of South Australia Human Ethics Committee and the governing recruitment site (CALHN). The study was performed in accordance with the ethical standards laid down in the 1964 Declaration of Helinski. All participants provided written informed consent.

### Participants

Potential participants admitted to the Royal Adelaide Hospital (RAH) stroke unit were screened for inclusion. The inclusion criteria were: a confirmed diagnosis of stroke on computed tomography and/or magnetic resonance imaging, recruited within 1–14 days of stroke, medically stable, able to provide informed consent (or legal guardian to provide third party consent), any impairment/s (weakness, altered sensation, loss of dexterity, reduced coordination) and ≥18 years of age. Exclusion criteria were patients deemed for palliative or comfort care, an inability to communicate in English (unless a family member was present to interpret) or receptive/expressive aphasia that would interfere with testing. Consecutive recruitment was performed by screening all patients on the Stroke unit daily. With no previous studies to guide sample size calculations and given the exploratory nature of this study, the aim was to maximise recruitment within a period of 8 months.

### Outcome Measures

The assessment battery included key outcomes to characterise the cohort [baseline only; National Institutes of Health Stroke Scale (NIHSS), the Montreal Cognitive Assessment (MOCA) and Functional Independence Measure (FIM)] along with repeated assessments at baseline, 1, 3 and 6 months following stroke for sensation, body awareness, self-efficacy, quality of life and motor impairment/function.

Body awareness was assessed with the Body Perception Disturbance (BPD; UL and LL) (measures physical awareness of limb ownership, awareness of limb position, attention required to attend to limb, emotional feelings toward limb, difference in size, temperature, pressure, weight, and the description/mental image of body parts) and the Multidimensional Assessment of Interoceptive Awareness (MAIA) (measures interoceptive awareness, the perception of sensation from inside the body including noticing, distracting, worrying, attention regulation, emotional awareness, self-regulation, body listening and trusting). The MAIA was considered inappropriate to perform in acute phase as some questions were potentially distressing, therefore this measure was only performed at 1, 3, and 6 months after stroke.

Sensation was assessed using the Erasmus Nottingham Sensory Assessment for Upper Limb- EmNSA- UL [measures tactile-light touch, pressure, pinprick, temperature, tactile localisation, bilateral simultaneous touch), kinaesthetic, stereognosis].

Self-efficacy was assessed with the Stroke Self-Efficacy Questionnaire (SSEQ); quality of life with the Stroke Impact Scale (SIS) and Stroke Specific Quality of Life Scale (SSQoL); a comprehensive assessment of motor impairment after stroke using the Fugl-Meyer Upper Extremity (FMA-UE); and the Motor Activity Log (MAL) to observe the amount and quality of motor function. All participants were recruited and assessed within the 14 days following stroke admission. All assessments were performed by a trained and experienced therapist (IS) and all participants received standard stroke and rehabilitation care.

### Data Analysis

Statistical tests were performed in IBM SPSS Statistics 25 with level of significance set at *p* < 0.05. Data were tested for normality using the Shapiro-Wilk test, and where required, non-parametric tests were used. Descriptive statistics were used to report the mean and standard deviation of clinical characteristics. Linear mixed effects models were used to test for the fixed effect of VISIT (change over time at four assessment points) for all outcomes including sensation (EmNSA), body awareness (BPD, MAIA), self-efficacy (SSEQ) quality of life (SIS, SSQoL) and motor function (FMA, MAL). The estimated means and standard error of the measure at each time point (baseline, 1, 3, and 6 months) were calculated. Where appropriate, pairwise comparisons, adjusting for multiple comparisons using Bonferroni corrections, were then performed to calculate the mean difference, standard error and significance at each time point. The Spearmans rank test (non-parametric) was used to explore bivariate correlations: if body awareness was associated with stroke recovery in terms of sensation, motor impairment and quality of life. As a preliminarily step to investigate possible associations between body awareness and sensation with patient demographics (age, gender, time since stroke, side affected) and clinical outcomes (NIHSS, MOCA, FIM, SSEQ, SIS, SSQoL, MAL, FMA), correlations were performed by averaging clinical outcomes over time.

## Results

### Participants

**Figure 1:**
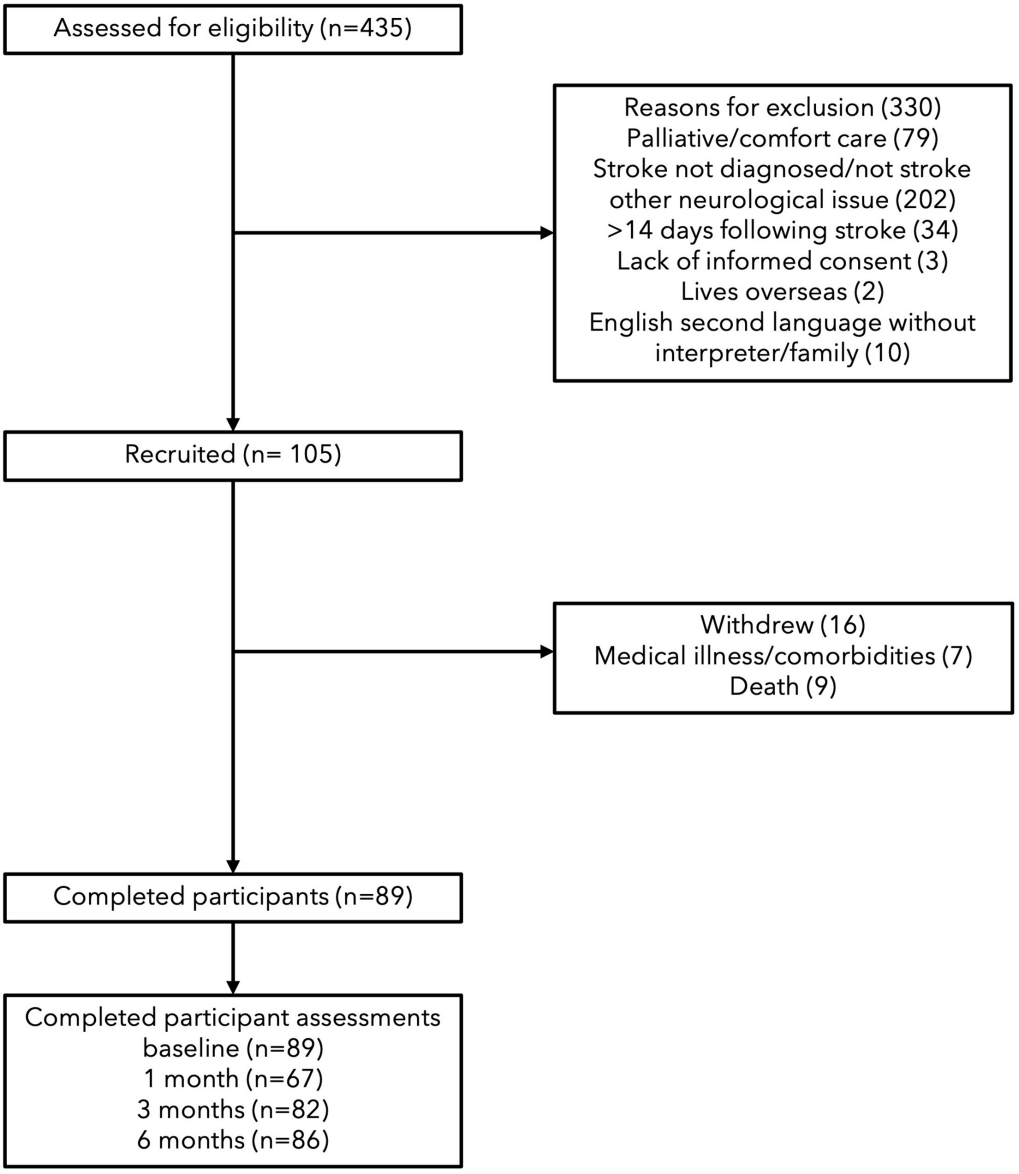
STROBE flow diagram of observational study.

A total of 105 participants were recruited, 16 were withdrawn before baseline testing (data not included) due to medical illness or death; with 89 continuing until completion. Participants were aged between 45 and 93 years and were first assessed between zero to 11 days (see Table 1). Thrombolysis rates at the recruiting hospital are on average 17% and this sample would reflect that as it is representative (57). At 1 month, 67 participants were tested, followed by 82 at 2 months and 86 at 6 months (see Figure 1). The increase in missing assessments at 1 month were due to loss of contact in transition from hospital to rehabilitation, transitional care or home, *n* = 22. In relation to sensation and body awareness, half of the participants exhibited proprioceptive and tactile impairments, while all participants showed impairments in interoceptive awareness.

**Table 1:** Baseline characteristics.

**Characteristic**	**Participants (*n* = 89)**
Age (year), mean (SD)	71.9 (12.1)
Gender, *n* (%) female	38 (43%)
TSS to baseline assessment (days), mean (SD)	3.6 (2.1)
Side affected, *n* (%) left	58 (65%)
Stroke type, *n* (%) ischaemic	77 (86.5%)
Premorbid residence (metropolitan), *n* (%)	56 (63%)
NIHSS, mean (SD)	7.8 (5.7)
MOCA, mean (SD)	21.4 (5.9)
FIM, mean (SD)	74.1 (23.1)
MAIA, *n* (%) interoception affected	89 (100)

*TSS, time since stroke; NIHSS, National Institutes of Health Stroke Scale; MOCA, Montreal Cognitive Assessment; FIM, Functional Independence Measure*.

### Change Over Time Comparisons

**Figure 2:**
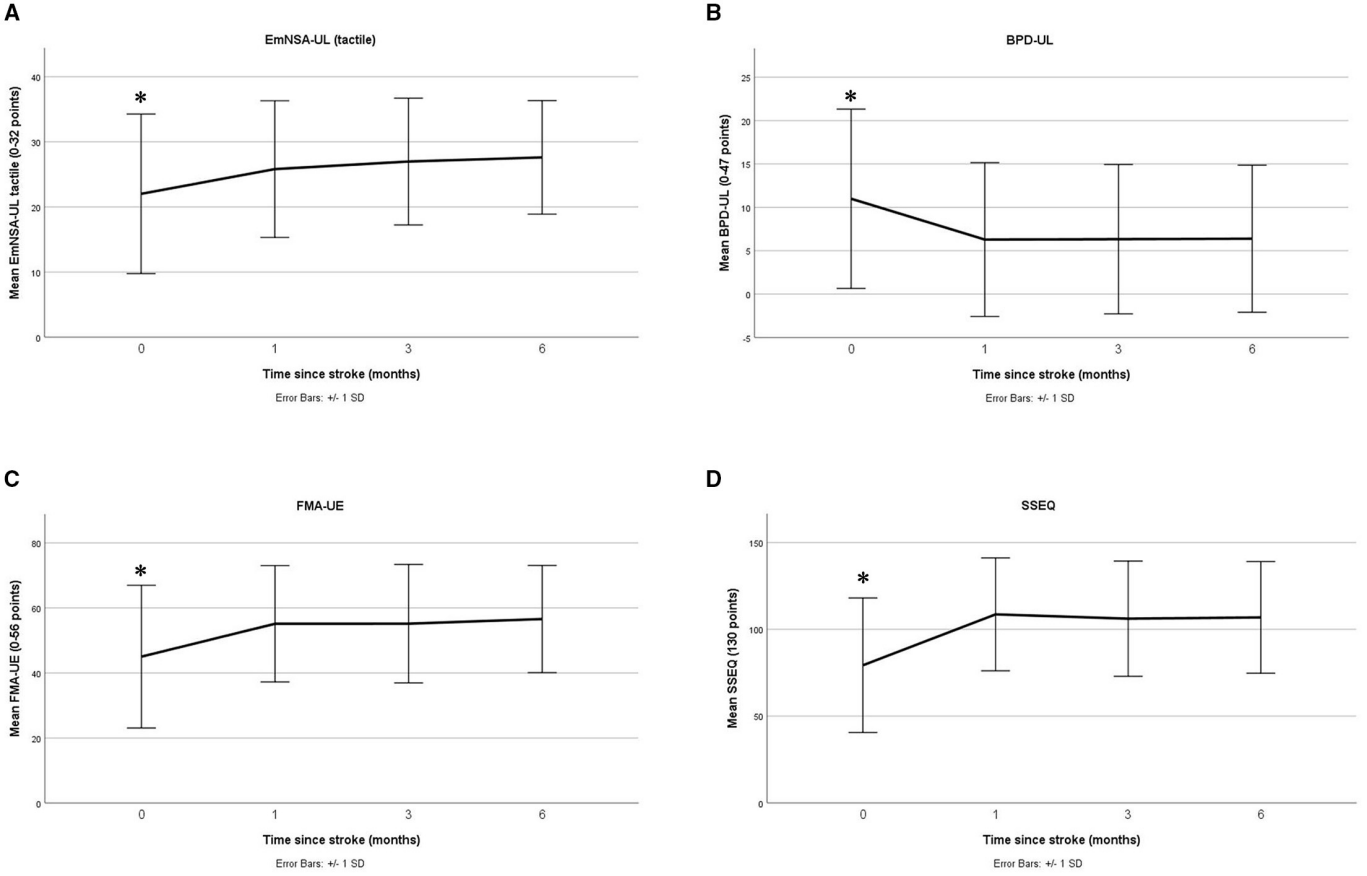
Change over time (mean and standard deviation) for sensation **(A)**, body awareness **(B)**, motor impairment **(C)**, and quality of life **(D)** (baseline to 6 months) * indicates *post-hoc* analysis found significant differences at this timepoint.

Each linear mixed model investigating recovery outcomes over time reached significance (all *p* < 0.05, see Table 2) except for the MAIA where a significant change over time was not observed (*p* = 0.205) (note, for ethical reasons this measure was not recorded at baseline). In general, for the outcomes that did reach statistical significance, a marked change was observed between baseline and one month, with a plateau between months 1 and 3 (see Figure 2 and *post-hoc* comparisons in Appendix 1).

**Table 2:** Change over time for recovery measures (baseline to 6 months).

	**Sensation**		**Body awareness**			**Self-efficacy**	**Quality of life**				**Motor impairment/function**	
**Measure (mean, SE) Timepoint**	**EmNSA-UL Tactile**	**EmNSA-UL Proprio**	**BPD-UL**	**BPD-LL**	**MAIA**	**SSEQ**	**SIS total**	**SIS %**	**SSQOL**	**MAL QOM**	**MAL AOU**	**FMA- UE**
Baseline	22.0 (12.3)	6.36 (2.90)	11.0 (10.3)	7.41 (9.20)	–	79.3 (38.8)	174.9 (31.8)	53.2 (25.6)	150.7 (33.9)	1.80 (3.77)	1.88 (3.85)	45.0 (22.0)
1 month	25.8 (10.5)	7.60 (1.43)	6.28 (8.86)	3.91 (7.25)	14.1 (6.34)	108.6 (32.5)	225.0 (45.5)	71.5 (20.0)	190.4 (38.4)	3.20 (1.84)	3.23 (1.87)	55.1 (17.9)
3 months	27.0 (9.74)	7.60 (1.50)	6.33 (8.61)	4.66 (7.85)	13.7 (6.25)	106.2 (33.2)	226.1 (46.3)	77.1 (35.6)	189.9 (46.1)	3.29 (1.85)	3.32 (1.88)	55.2 (18.2)
6 months	27.6 (8.72)	7.57 (1.52)	6.38 (8.48)	4.79 (7.22)	13.5 (6.22)	106.9 (32.2)	227.7 (47.4)	74.7 (25.7)	193.9 (43.8)	3.30 (1.79)	3.30 (1.82)	56.6 (16.5)
*p*-value	<0.001^*^	<0.001^*^	<0.001^*^	<0.001^*^	0.205	<0.001^*^	<0.001^*^	<0.001^*^	<0.001^*^	0.002^*^	<0.001^*^	<0.001^*^

*BPD, Body Perception Disturbance Scale; EmNSA UL tactile, Erasmus modified Nottingham Sensory Assessment-Upper Limb; FMA-UE, Fugl-Meyer Assessment- Upper Extremity; MAIA, Multidimensional Assessment of Interoceptive Awareness Questionnaire; MAL QOM/AOU, Motor Activity Log- Quality of Movement/Amount of Use; Proprio. (proprioception); SSEQ, Stroke Self-Efficacy Questionnaire; SIS, Stroke Impact Scale; SSQoL, Stroke-Specific Quality of Life Scale*;

* *significant p ≤ 0.05*.

### Correlation of Body Awareness and Sensation With Other Variables

Correlation analyses found that higher scores on sensation and body awareness measures were associated with improved clinical outcomes (see Table 3). Briefly, correlational analyses found upper limb body awareness (BPDUL) correlated strongly with lower limb body awareness (BPULL), self-efficacy (SSEQ), quality of life (SIS and SSQoL), and motor function/impairment (MALQQM, MALAOU, FMAUE). However, the MAIA body awareness measure showed very weak to weak correlations with body awareness (rho = −0.185 for BPDUL and rho = −0.156 for BPDLL) as well as other recovery measures. This was similar for EmNSA (tactile and proprioception), a measure of sensation which showed very weak to moderate correlations with all body awareness measures (BPDUL, BPDLL and MAIA), and this was further seen with the other recovery measures.

**Table 3:** Correlational analyses of sensation and body awareness with other recovery measures (Spearmans rho).

	**Sensation**		**Body Awareness**		
**Parameter**	**EmNSA (Tactile)**	**EmNSA (Proprio.)**	**BPD UL**	**BPD LL**	**MAIA**
Gender (M/F)	0.206^**^	0.065	−0.040	−0.138^*^	0.063
Age (years)	0.048	−0.090	0.094	0.076	−0.143^*^
Time since stroke (days)	0.090	0.040	−0.270^**^	−0.252^**^	−0.162^*^
Side affected (L/R)	−0.060	−0.091	−0.028	−0.073	−0.074
NIHSS	−0.269^**^	−0.084	0.384^**^	0.191^**^	−0.049
MOCA	0.064	0.074	−0.266^**^	−0.174^*^	0.318^**^
FIM	0.111^*^	0.085	−0.444^**^	−0.241^**^	0.057
Location (metropolitan/rural)	0.236^**^	−0.001	0.062	0.111^*^	−0.186^**^
EmNSA Tactile	**1.000**	0.517^**^	−0.288^**^	−0.188^**^	0.073
EmNSA Proprio.	0.517^**^	**1.000**	−0.228^**^	−0.135^*^	0.177^*^
BPDUL	−0.288^*^	−0.228^**^	**1.000**	0.765^**^	−0.187^**^
BPDLL	−0.188^**^	−0.135^*^	0.765^**^	**1.000**	−0.156^*^
MAIA	0.073	0.177^*^	−0.187^**^	−0.156^*^	**1.000**
SSEQ	0.254^**^	0.236^**^	−0.666^**^	−0.513^**^	0.206^**^
SIS Total	0.301^**^	0.322^**^	−0.696^**^	−0.516^**^	0.210^**^
SIS %	0.334^**^	0.219^**^	−0.583^**^	−0.436^**^	0.130
SSQoL	0.266^**^	0.297^**^	−0.658^**^	−0.480^**^	0.194^**^
MALQOM	0.328^**^	0.267^**^	−0.723^**^	−0.493^**^	0.170^*^
MALAOU	0.313^**^	0.283^**^	−0.702^**^	−0.481^**^	0.193^**^
FMAUE	0.324^**^	0.260^**^	−0.661^**^	−0.440^**^	0.214^**^

*Correlations: 0.00–0.19 very weak; 0.20–0.39 weak; 0.40–0.59 moderate; 0.60–0.79 strong (bold/underlined); 0.80–1.00 very strong (bold/underlined)*;

* *(correlation is significant at the 0.05 level)*;

** *(significant the 0.01 level)*.

*BPD, Body Perception Disturbance Scale; EmNSA UL tactile, Erasmus modified Nottingham Sensory Assessment-Upper Limb; FMA-UE, Fugl-Meyer Assessment- Upper Extremity; MAIA, Multidimensional Assessment of Interoceptive Awareness Questionnaire; MAL QOM/AOU, Motor Activity Log- Quality of Movement/Amount of Use; Proprio. (proprioception); SSEQ, Stroke Self-Efficacy Questionnaire; SIS, Stroke Impact Scale; SSQoL, Stroke-Specific Quality of Life Scale*.

*Gender: Male = 1; Female = 2; Side affected: Left = 1, Right = 2; Location: Metropolitan = 1; Rural = 2*.

## Discussion

### Main Findings

To our knowledge, this is the first study to explore impairment of body awareness following stroke and the associated recovery across subsequent months. The main finding of this study was that body awareness was reduced after stroke and improved within the first month, but plateaued with post-stroke gains persisting between 1 and 3 months. This was similar for measures of sensation, motor impairment, function, self-efficacy and quality of life. In addition, greater body awareness was associated with better outcomes for the five mentioned key recovery domains. This is an important finding because it provides insight into understanding the importance of body awareness after stroke. Further, this pattern fits typical stroke and recovery processes. Preclinical studies investigating motor recovery highlight that neural plasticity is enhanced early after stroke, both facilitating and accelerating recovery (56). In humans, behavioural studies suggest motor recovery predominantly occurs within the first 3–6 months before improving further, plateauing or even declining (54, 56). Our current findings suggest a similar pattern may emerge for body awareness (54). These findings are in line with literature concerning other post stroke impairments, possibly because this period of rapid stroke recovery is linked with spontaneous upregulation of plasticity (54). Most recovery was observed within 1 month, this coincides with heightened plasticity and likely delivery of rehabilitation services (54, 56). Alternatively, cost, availability and type of therapeutic input available subsequently required to maintain sufficient levels of therapy dosage may be inadequate.

These findings from this paper highlight the potentially important association that body awareness (using the BPD measure of the upper limb) in particular may have with motor function/impairment (MAL and FMA-UE), self-efficacy (SSEQ) and quality of life (SIS and SSQoL). Although the BPD measure was subjective, the questions directly related to body awareness impairments commonly experienced after stroke and were rated on a numerical scale. It was also time efficient to apply and directly related to symptoms of body awareness post-stroke such as limb size, weight and position (notably both body schema and body image). That the upper limb section appeared to have stronger correlations with other clinical outcomes compared to the lower limb section of the BPD might be explained by the arm having a greater prevalence of sensory loss post-stroke (43, 45, 50). Although other factors beyond sensorial are thought to contribute to body awareness, unexpected findings were found with a poor association between body awareness using the MAIA measure, and all other recovery measures. This may be because this measure was only assessed from 1 to 6 months and did not include a baseline assessment, or perhaps it may not be a useful measure for stroke. Further, the complexity of the questions (e.g., on a scale of zero to five how often do I “listen to my body to inform me about what to do”) may have reduced the accuracy of the responses. It should be noted that the MAIA is only validated in the pain and anxiety literature, and may not translate to this stroke population. Even more surprisingly both measures of body awareness were poorly associated with sensation despite the conceptual models indicating sensation and body awareness are related (10, 41). This finding might suggest body awareness impairments are only partly explained by sensory loss and other perceptual and conceptual processes are more likely to contribute.

A few studies have looked at whether implementing a body awareness training program improves motor impairment and function, however none of these studies measured body awareness (51–53, 58, 59). Only one recent study has looked at this relationship in stroke and reported a positive association between body awareness, balance function (postural control) and independence in activities of daily living (41). This study showed a high level of body awareness was observed in the group with high functional (balance) abilities and independence in daily activities. The focused attention on both the performance and experience during the movements increased the physical and mental aspects of body awareness. Unfortunately, no reports of self-efficacy have yet been assessed (41).

### Strengths and Limitations

This study had several strengths including a large sample size; consecutive (representative) case recruitment; heterogeneity in participant demographics and severity of impairments; and the number of follow-up assessments, as well as face validity for the usefulness of the Body Perception Disturbance measure in individuals following stroke. For the limitations of this study we acknowledge not all participants were available at follow-up assessments for a variety of reasons. Therefore, results should be viewed cautiously. The inclusion of a 12-month assessment to review the longer-term pattern of recovery, as well as the inclusion of a baseline neglect measure also may prove beneficial. In addition, all participants were recruited from one main hospital in South Australia potentially reducing the generalisability of this study's findings. Therapy (including thrombolytics) received during the first month and post-discharge from the acute settings was not individually documented and may have been useful to further understand and clarify these findings. Lastly, the BPD (60) and MAIA (1) body awareness measures were sourced from non-stroke literature (pain and anxiety, respectively) because stroke-validated tests could not be identified (in press); therefore they provide only an indication of the importance and impact of body awareness post-stroke.

### Future Directions

Future studies should consider lesion characteristics which may help further understand the effects of stroke on body awareness, as well as the relative vulnerability of relevant networks to stroke. A body awareness measure that assesses all constructs of body awareness (image and schema) appropriately for stroke now needs to be developed and piloted in both healthy and stroke populations. The BPD measure appears to have constructs that are most relevant in the stroke population as it covers several aspects of body awareness including body schema and some structural features. However, we do not recommend the MAIA for several reasons: some of the questions in the MAIA were felt to be too distressing for someone in the acute stroke phase, and it also includes hypervigilance (as a result of anxiety) which is not relevant in stroke where hypo-vigilance and neglect are more likely scenarios. In addition the inclusion of neurological imaging could confirm the hypothesised networks regarding body schema and body image.

A further large prospective study is now required to replicate our findings and advance our understanding of the drivers of body awareness, to more definitively understand the role it may play in motor impairment and function, and to develop effective assessment and intervention strategies. The current findings open the door to develop a greater understanding of how body awareness specifically changes following a stroke and how these are modified by the passage of time. It also allows consideration of new treatment approaches such as interventions that might seek to enhance body awareness directly.

## Conclusion

In summary, this is the first study to document changes in body awareness over time and its association with other key stroke recovery outcomes. We observed body awareness was reduced after stroke, appeared to recover somewhat within the first month and was correlated with clinical outcomes for self-efficacy, quality of life and motor function/impairment. These findings highlight the importance that body awareness may play in stroke recovery. Such information might enable clinicians to intervene more effectively to facilitate recovery either by improving awareness in order to improve activities of daily living or by teaching individuals to find an effective alternative for these difficulties.

## Data Availability Statement

The original contributions presented in the study are included in the article/Supplementary Material, further inquiries can be directed to the corresponding author/s.

## Ethics Statement

The studies involving human participants were reviewed and approved by University of South Australia Human Ethics Committee and the Central Adelaide Local Health Network (CALHN). The patients/participants provided their written informed consent to participate in this study.

## Author Contributions

All authors listed have made a substantial, direct, and intellectual contribution to the work and approved it for publication.

## Conflict of Interest

The authors declare that the research was conducted in the absence of any commercial or financial relationships that could be construed as a potential conflict of interest.

## Publisher's Note

All claims expressed in this article are solely those of the authors and do not necessarily represent those of their affiliated organizations, or those of the publisher, the editors and the reviewers. Any product that may be evaluated in this article, or claim that may be made by its manufacturer, is not guaranteed or endorsed by the publisher.
