# Supplementary material for: Recovery of Body Awareness After Stroke: An Observational Study
Source: Front Neurol. 2021 Nov 29;12:745964. doi: 10.3389/fneur.2021.745964 (PMC8666978; doi:10.3389/fneur.2021.745964)
Supplement: Supplementary file 1 [file Table_1.DOCX]

Appendix 1 | Post-hoc comparisons- significant interactions between each time point

|  |  | Sensation | | Body Awareness | | | Self-efficacy | Quality of Life | | | | | | | Motor Impairment/ Function | | | | | | |
| --- | --- | --- | --- | --- | --- | --- | --- | --- | --- | --- | --- | --- | --- | --- | --- | --- | --- | --- | --- | --- | --- |
| Timepoint | | EmNSA UL Tactile | EmNSA Proprio. | BPD-UL | BPD-LL | MAIA | SSEQ | SIS total | | SIS % | | SSQOL | | MAL QOM | | | MAL AOU | | FMA-UE | |  |
| Baseline | 1m | 0.001* | 0.001* | 0.001* | 0.001* | - | 0.001* | 0.001* | | 0.001* | | 0.001* | | 0.002* | | | 0.006* | | 0.001* | |  |
|  | 6m | 0.001* | 0.001* | 0.004 | 0.318 | - | 0.001* | 0.001* | 0.001* | | 0.001* | | 0.001* | | | 0.004* | | 0.001* | |  |  |
| 1 month | 3m | 0.226 | 0.838 | 1.000 | 0.261 | 1.000 | 0.967 | 1.000 | 0.566 | | 1.000 | | 0.412 | | | 0.193 | | 1.000 | |  |  |
| 3 months | 6m | 1.000 | 1.000 | 1.000 | 0.923 | 0.371 | 1.000 | 1.000 | 0.779 | | 1.000 | | 1.000 | | | 0.384 | | 1.000 | |  |  |
| Abbreviations: BPD (Body Perception Disturbance Scale); EmNSA UL tactile (Erasmus modified Nottingham Sensory Assessment-Upper Limb); FMA-UE (Fugl-Meyer Assessment- Upper Extremity); m (month/s); MAIA (Multidimensional Assessment of Interoceptive Awareness Questionnaire); MAL QOM/AOU (Motor Activity Log- Quality of Movement/Amount of Use); Proprio. (proprioception); SSEQ (Stroke Self-Efficacy Questionnaire); SIS (Stroke Impact Scale); SSQoL (Stroke-Specific Quality of Life Scale); * (significant if p≤0.05) | | | | | | | | | | | | | | | | | | | | | |
